# A new perspective on fungal metabolites: identification of bioactive compounds from fungi using zebrafish embryogenesis as read-out

**DOI:** 10.1038/s41598-019-54127-9

**Published:** 2019-11-26

**Authors:** Jelmer Hoeksma, Tim Misset, Christie Wever, Johan Kemmink, John Kruijtzer, Kees Versluis, Rob M. J. Liskamp, Geert Jan Boons, Albert J. R. Heck, Teun Boekhout, Jeroen den Hertog

**Affiliations:** 1 Hubrecht Institute – KNAW and University Medical Center Utrecht, Utrecht, The Netherlands; 2 Utrecht University, Chemical Biology & Drug Discovery, Utrecht Institute for Pharmaceutical Sciences, Utrecht, The Netherlands; 3 Faculty of Science and Engineering, University of Groningen, Groningen, The Netherlands; 4 Utrecht University, Biomolecular Mass Spectrometry and Proteomics, Bijvoet Center for Biomolecular Research and Utrecht Institute for Pharmaceutical Sciences, Utrecht, The Netherlands; 5 Present Address: School of Chemistry, University of Glasgow, Glasgow, UK; 6 Westerdijk Institute for Fungal Biodiversity - KNAW, Utrecht, The Netherlands; 7 Institute of Biodynamics and Ecosystem Dynamics, University of Amsterdam, Amsterdam, The Netherlands; 8 Institute Biology Leiden, Leiden University, Leiden, The Netherlands

**Keywords:** Developmental biology, Screening

## Abstract

There is a constant need for new therapeutic compounds. Fungi have proven to be an excellent, but underexplored source for biologically active compounds with therapeutic potential. Here, we combine mycology, embryology and chemistry by testing secondary metabolites from more than 10,000 species of fungi for biological activity using developing zebrafish (*Danio rerio*) embryos. Zebrafish development is an excellent model for high-throughput screening. Development is rapid, multiple cell types are assessed simultaneously and embryos are available in high numbers. We found that 1,526 fungal strains produced secondary metabolites with biological activity in the zebrafish bioassay. The active compounds from 39 selected fungi were purified by liquid-liquid extraction and preparative HPLC. 34 compounds were identified by a combination of chemical analyses, including LCMS, UV-Vis spectroscopy/ spectrophotometry, high resolution mass spectrometry and NMR. Our results demonstrate that fungi express a wide variety of biologically active compounds, consisting of both known therapeutic compounds as well as relatively unexplored compounds. Understanding their biological activity in zebrafish may provide insight into underlying biological processes as well as mode of action. Together, this information may provide the first step towards lead compound development for therapeutic drug development.

## Introduction

Due to globalization, an ageing world population and increasing resistance to existing drugs, there is a need for the development of new drugs. Only a relatively low number of biologically active compounds are being used in prescription drugs. Furthermore, these biologically active compounds are often chemically related to each other^1^. Hence, it is important to uncover new biologically active molecules which have the potential to be developed further into drugs. Despite great efforts to generate synthetic drugs, only 27% of the approved drugs in the clinic are of completely synthetic origin^2^. Most of the drugs that are currently being used in the clinic are derived from natural products.

Fungi are a rich source of biologically active natural compounds. They produce a plethora of biologically active secondary metabolites, including a wide variety of clinically important drugs. For example, fungi produce beta lactam antibiotics such as penicillin and cephalosporin, which have been dominating the antibiotic market until recently, and were the stepping stones for the development of next generation antibiotics^3^. Furthermore, other fungal compounds such as the immunosuppressant cyclosporine and cholesterol lowering compactin and lovastatin are frequently used in the clinic^3–5^. Given the estimates of the biodiversity in the fungal kingdom and the notion that only a small fraction of fungi has ever been tested for the production of biologically active compounds, it is evident that a wealth of compounds is still to be discovered. This is underlined by the identification of numerous new fungal metabolites in recent years^6–10^.

To uncover compounds with biological activity produced by fungi, we use zebrafish as a model system. This has several advantages over other systems. To assess biological activity in higher eukaryotes, tissue culture cells are often being used. However, this limits screens to cell type specific effects. In order to do whole animal testing, zebrafish (*Danio rerio*) embryos are a great model. Zebrafish are vertebrates with a highly conserved physiology. The embryos develop in aqueous medium and readily take up compounds from the medium. Furthermore, the embryos are transparent and develop all primary organs and tissues in a few days. This makes biological activities of compounds readily detectable as developmental defects in zebrafish embryos. In addition, due to the high fecundity of zebrafish, large numbers of embryos can be obtained for experiments relatively easily, which makes them an excellent model for high-throughput screens^11,12^. To date, over 65 small-molecule screens using zebrafish have been reported with various read-outs such as embryo morphology or behavioral differences^13^. These screens have been successful in either discovering novel compounds or finding new purposes for existing drugs^14–16^. In the process, phenotypes caused by existing drugs may be indicative for the mode-of-action or targets of unexplored compounds.

Here, we combined mycology, embryology and chemistry in our analysis of 10,207 fungal species from the large collection of the Westerdijk Fungal Biodiversity Institute. We screened these fungi for production of secondary metabolites with biological activity, using zebrafish embryogenesis as read-out. We found that over 15% of the fungi produced secondary metabolites which affected embryonic development. The phenotypes caused by these secondary metabolites are diverse, ranging from severe pleiotropic to highly specific phenotypes. We selected 39 fungi for further analysis from which we purified and identified 34 metabolites which induced specific developmental defects in zebrafish embryos. This group of identified metabolites consists of both known therapeutic compounds as well as relatively unexplored compounds that induced similar phenotypes as well-known therapeutic compounds. In the process, we generated a library of secondary metabolites from 10,207 fungi which can be tested in any bioassay.

## Results

### Generation of a library of secreted secondary metabolite mixtures from 10,207 strains of fungi

**Figure 1:**
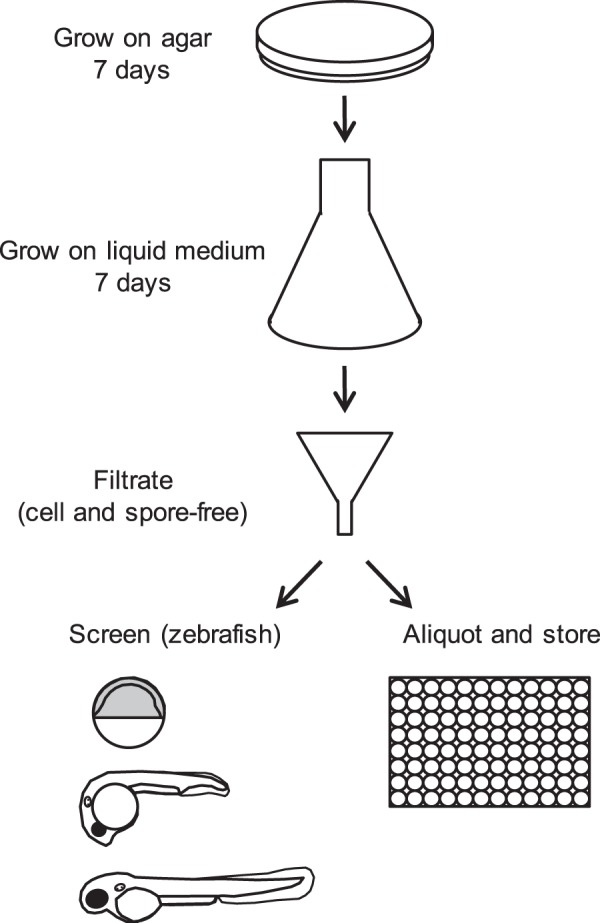
Work-flow of the generation of the library of secondary metabolite mixtures from 10,207 strains of fungi and initial screen using zebrafish embryos as a read-out. See text for details.

A library of secreted secondary metabolites mixtures was generated using 10,207 strains of fungi as outlined in Fig. 1. In short, lyophilized fungal strains were suspended in malt peptone and inoculated on agar plates. In case of sufficient growth, liquid medium aliquots (3.5% Czapek Dox broth + 0.5% Yeast extract) were inoculated with the fungi. This medium showed the best results in a pilot experiment using a variety of liquid media on a selection of fungi. After seven days, the broth was filtered to remove fungi and spores, resulting in sterile filtrates containing secondary metabolites. The fungal strains that were used to generate this library were picked from the collection of the Westerdijk Institute in an unbiased manner. A complete list of the 10,207 fungi used is included in the Supplementary Table S1.

### Screen of zebrafish embryos

To assess biological activity of the fungal filtrates, five zebrafish embryos at 6 hours post fertilization (hpf) in E3 medium were incubated simultaneously with fungal filtrates (1:1, v-v) at 28 °C. At developmental stages 24 hpf and 48 hpf, the zebrafish embryos were inspected for morphological developmental defects. If 3 or more of the 5 embryos displayed similar developmental defects after repeated testing, the fungal filtrate was scored as positive. In addition, in case incubation with fungal filtrates was toxic, resulting in embryonic lethality at 24 or 48 hpf, the fungal filtrates were tested in serial dilutions until an ineffective dilution was reached.

**Figure 2:**
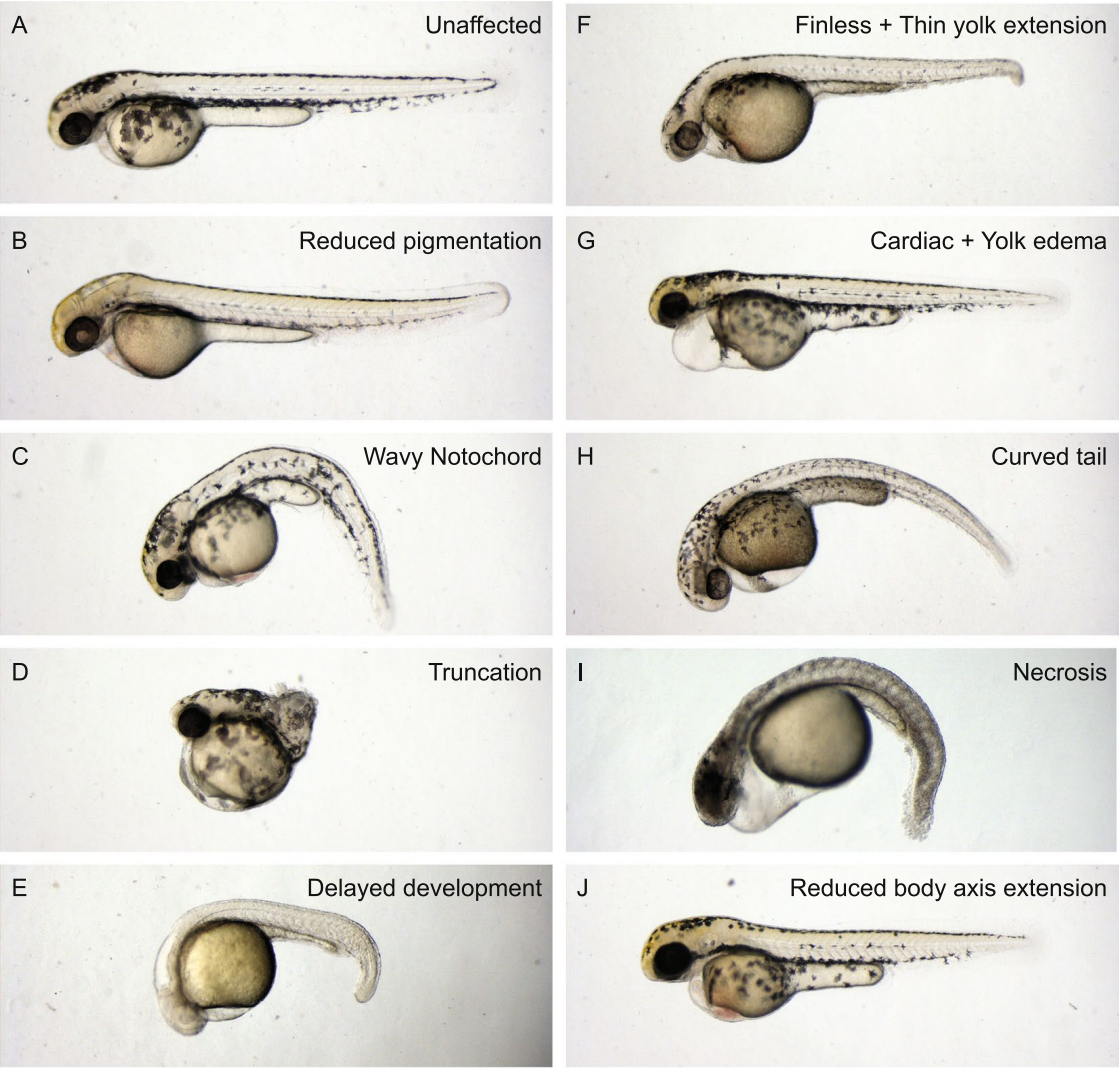
Fungal secondary metabolite mixtures induced distinct morphological defects in zebrafish embryos. Zebrafish embryos were incubated with fungal secondary metabolite mixtures from 6 hpf onwards. Embryos were imaged at 48 hpf. (**A**) Control not incubated with a fungal secondary metabolite mixture. (**B–H**) Examples of developmental defects caused by distinct fungal secondary metabolite mixtures. Note the diversity in developmental defects between samples.

Morphological defects were assessed by phase contrast microscopy. Embryos were visually inspected and all differences from wild type morphology were scored. Pictures were taken of treated embryos during the screen to document developmental defects. In total, 1526 fungal filtrates (14,95%) induced defects in zebrafish embryos. We classified the phenotypical defects in 13 distinct categories: pigmentation, notochord, truncation, delayed development, fin, tail, heart, yolk, yolk extension, body axis extension, necrosis, death and other. These categories will be further explained below. In 464 cases, phenotypes were assigned to more than one category. Table 1 shows how many filtrates induced a particular phenotype. Representative examples of developmental defects induced by incubation with fungal filtrates are depicted in Fig. 2.

**Table 1:** Phenotype categories and number of positives

Observed defects	Total	Unique
Death	551	551
Pigmentation	79	18
Notochord	14	11
Truncation	67	56
Delayed development	56	35
Fins	50	6
Heart	435	110
Yolk	291	14
Yolk extension	65	4
Tail	385	64
Necrosis	148	88
Body axis extension	105	13
Other	136	92
Combination of phenotypes	-	464
Total		1526

Of the 1526 fungal filtrates that induced morphological developmental defects in zebrafish embryos, 551 were categorized in the non-specific “death” category. The filtrates were lethal in the initial concentration. Further dilution of these fungal filtrates abolished the lethal defects, but did not lead to any specific developmental defect. In 79 cases we observed pigmentation defects. Most commonly, this involved less or no melanocytes compared to the untreated wild type embryo (Fig. 2B). Upon dilution of the fungal filtrate, the pigmentation gradually increased to normal. A rare but very specific phenotype is defective notochord development (14 times observed). Characteristically, this category involved an undulating or kinked notochord and a curved tail (Fig. 2C). Truncated embryos (67 times observed) lack formation of a tail, although the anterior part of the embryo developed relatively normally (Fig. 2D). Upon dilution of the sample, the truncation effect decreased and embryos developed a curved tail and reduced body axis extension was observed. Another interesting category is delayed development (56 times observed). Here, embryonic development is slowed down or even stalled when embryos are incubated with fungal filtrate, causing them to resemble an earlier developmental stage (Fig. 2E). We found less or no fin formation on 50 occasions. This phenotype was often accompanied by a thin yolk extension (Fig. 2F). The largest category of specific defects was heart defects (435 times observed). This category covers all phenotypes involving the cardiac area. Typically, heart defects are accompanied by a cardiac edema, a stretched heart and malfunctioning blood circulation (Fig. 2G). This phenotype is commonly seen in combination with other phenotypes, including yolk defects. Yolk defects (291 times observed) mostly involved yolk edemas or yolk hemorrhages that mostly occurred simultaneously with cardiac defects and seldom on their own. In other cases, we observed a change of color of the yolk, caused by colored compounds in the medium or necrosis. We intentionally included a separate category for yolk extension defects. In these cases, the yolk itself appears normal, but the yolk extension is either swollen, thinner or even completely absent (65 times observed). Again, this phenotype rarely occurred on its own and was mostly observed in combination with either fin or tail defects (Fig. 2F). The second biggest specific category is tail defects (358 times), covering all phenotypes involving the tail area, but excluding notochord defects and truncation phenotypes. The embryo depicted in Fig. 2H suffered from a characteristic curved tail, as seen in 108 cases. Necrosis, in either a part or the entire embryo, was observed with 149 filtrates (Fig. 2I). The final specific category was embryos with reduced body extension. At first glance embryos in this category look relatively normal. However, when measured they appear shorter. These developmental defects are reminiscent of the characteristic defects that result from defective convergence and extension cell movements during gastrulation (Fig. 2J). The “other” category involves all other effects caused by the fungal filtrates, including effects which were not necessarily developmental defects. For instance, several fungal filtrates dissolved the chorion or induced premature hatching of the zebrafish embryos without affecting the embryonic development itself.

### Purification and identification of biologically active compounds

**Figure 3:**
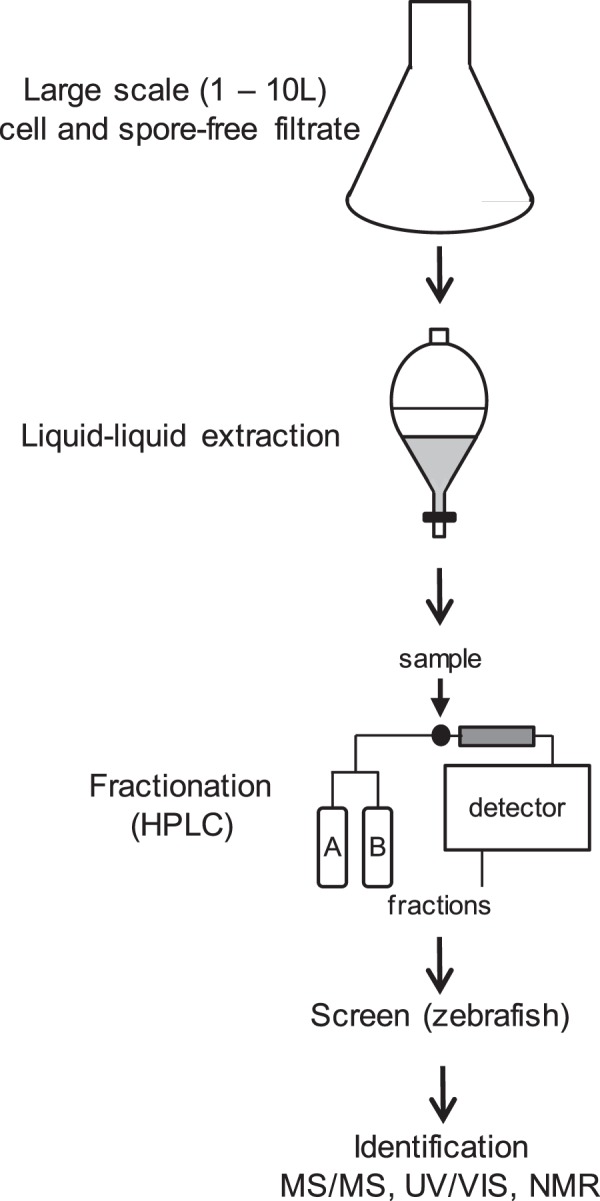
Schematic representation of the activity-guided purification and identification of active fractions. Briefly, fungi were grown at a large scale (1–10 L), extracted using ethyl acetate, evaporated to dryness and the residues separated using a preparative HPLC. Fractions were tested for activity using zebrafish embryonic development as a read-out and biologically active fractions were selected and subjected to spectroscopic identification and characterization methods. See Materials and Methods section for details.

To identify the mycotoxins in the fungal filtrates responsible for the developmental defects, we performed activity-guided purification as outlined in Fig. 3. First, fungi were grown at a large scale 1–10 l, and filtrated. Next, the fungal filtrates were extracted, using ethyl acetate, evaporated to dryness and the residues were dissolved in DMSO. Routinely, we tested the extracts for biological activity prior to further processing. In most cases the biological activity was successfully retained in the ethyl acetate extracts. Next, active extracts were fractionated using preparative HPLC. These fractions were tested on zebrafish, firstly, in pools of 6 fractions and subsequently, individual fractions of selected active pools were tested. Using this scheme, the individual fractions contained pure compounds in most cases, ready to be analyzed by analytical chemical methods. Typically, we determined the purity and nominal mass using LC-MS of the active compounds. Next, we determined the more accurate mass by high resolution mass spectrometry, UV/VIS spectra by spectrophotometry and overall molecular structure was elucidated by ^1^H-NMR and ^13^C-NMR. We compared the data with available literature and databases^17,18^, resulting in identification of previously identified compounds. For the identification of one compound, we performed 2D-NMR experiments, including Correlation Spectroscopy (COSY), Total Correlation Spectroscopy (TOCSY), Heteronuclear Single-Quantum Correlation Spectroscopy (HSQC) and Heteronuclear Multiple-Bond Correlation spectroscopy (HMBC).

### Proof of principle: identification of fusaric acid from *Fusarium proliferatum*

**Figure 4:**
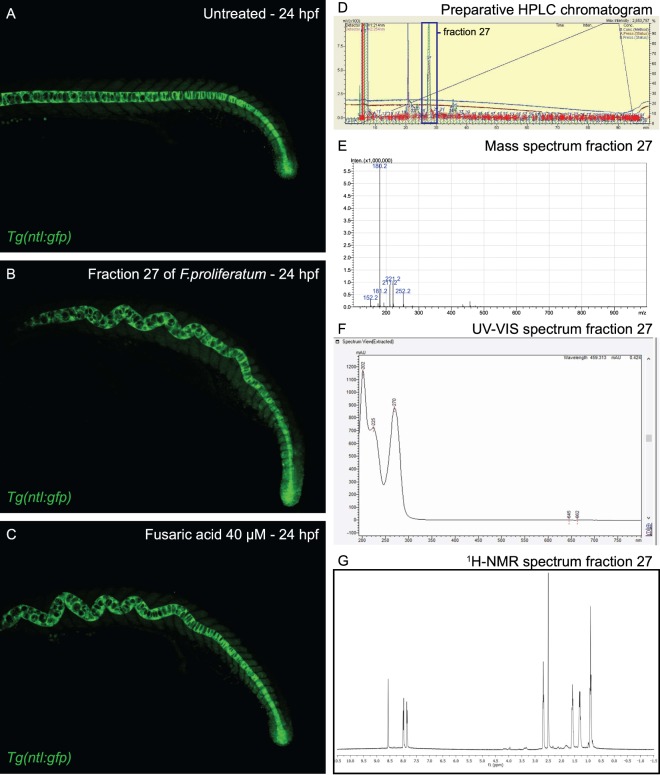
Identification of fusaric acid as bioactive compound from *Fusarium proliferatum* that induces an undulating notochord in zebrafish embryos. (**A**) *Tg(ntl-gfp)* transgenic zebrafish were left untreated or (**B**) were incubated with fraction 27 of *Fusarium proliferatum* and (**C**) fusaric acid (40 µM) from 6 hpf onwards and imaged at 24hpf using a confocal microscope, which highlights the undulating notochord in treated embryos. (**D–G**) Purification and identification of fusaric acid. (**D**) Preparative HPLC chromatogram of the secondary metabolite mixture of *fusarium proliferatum*. The major peak, fraction 27, contains the biologically active compound. (**E**) MS spectrum of fraction 27 revealing a M + H of 180.2 Da. (**F**) UV-Vis spectrum of fraction 27 revealing maximum absorption peaks at 202, 225 and 270 nm. (**G**) ^1^H-NMR spectrum of fraction 27.

We initially selected the fungal filtrate of *Fusarium proliferatum* (CBS 533.95), which induced a highly distinctive phenotype, an undulating notochord (Fig. 2C). The bioactive filtrate was extracted using ethyl acetate, the extract was dried, dissolved in DMSO and fractionated on a preparative HPLC. To illustrate the undulating notochord phenotype, we used transgenic *notail(ntl)-gfp* fish in which the gene encoding Green Fluorescent Protein (GFP) is knocked into the *ntl* gene, generating a Ntl-Gfp fusion protein which is expressed under the control of the *ntl* promoter^19^. Ntl-Gfp is expressed predominantly in the notochord in this transgenic line (Fig. 4A). Incubation of transgenic *ntl-gfp* embryos with fraction 27 of the fungal filtrate from *Fusarium proliferatum* induced an undulating notochord, which was illustrated spectacularly by fluorescence microscopy (Fig. 4B). The anterior half of the notochord undulates, whereas the posterior part of the notochord is straight, comparable to the control.

Fraction 27 of the bioactive filtrate from *Fusarium proliferatum* consisted of a single peak harboring the biological activity (Fig. 4D). Chemical analyses of this fraction resulted in identification of a compound with molecular mass of 179.2 Da (M + H = 180.2) (Fig. 4E), UV-absorption maxima of 202 nm, 225 nm and 270 nm (Fig. 4F) and a ^1^H-NMR spectrum (Fig. 4G). These data corresponded closely with reported data of fusaric acid^17^. Moreover, *Fusarium* species are well-known to produce fusaric acid^20^, and fusaric acid has been shown to induce an undulating notochord in zebrafish embryos^21^. Altogether, this suggested that the bioactive compound from *Fusarium proliferatum* was fusaric acid. To verify this, we tested commercially available fusaric acid in parallel with the bioactive compound we had isolated. The phenotypes induced by the purified compound and the commercially available fusaric acid were identical (Fig. 4C). Thus, we conclude that the compound produced by *Fusarium proliferatum* which induced an undulating notochord in zebrafish embryos was fusaric acid.

### Identification of secondary metabolites which induced shorter phenotypes

Next, we focused on the shorter category. Zebrafish embryos in this category display reduced body axis extension occasionally combined with other developmental defects, including cardiac edema, craniofacial defects, pigmentation defects and yolk defects (Fig. 2J). We selected this category because embryos in this category resemble embryos we have reported before, in which key signaling molecules have been knocked down or knocked out^22–26^. Furthermore, embryos which have been treated with small molecule inhibitors that induce convergence and extension cell movement defects, including the PI3Kinase inhibitor, LY294002^27,28^, show a similar phenotype. At later stages, this phenotype is combined with other defects, typically including cardiac edema, pigmentation defects and craniofacial defects. Interestingly, various signaling pathways are essential for normal convergence and extension cell movements, including non-canonical Wnt signaling and signaling by protein-tyrosine kinases/protein-tyrosine phosphatases^22–26,29,30^. We hypothesize that fungal compounds might act in these important pathways.

**Figure 5:**
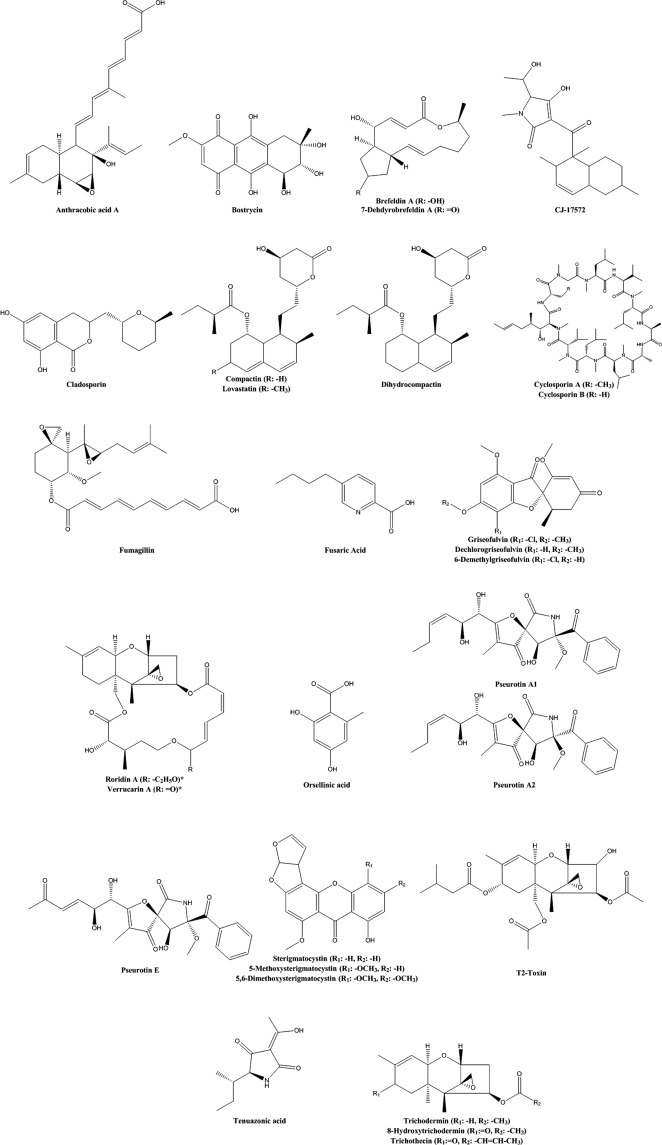
The chemical structures of compounds that reduce body axis extension are diverse. The chemical structures of the compounds that induce shorter phenotypes are depicted here. These compounds are listed in Table 2 as well.

**Figure 6:**
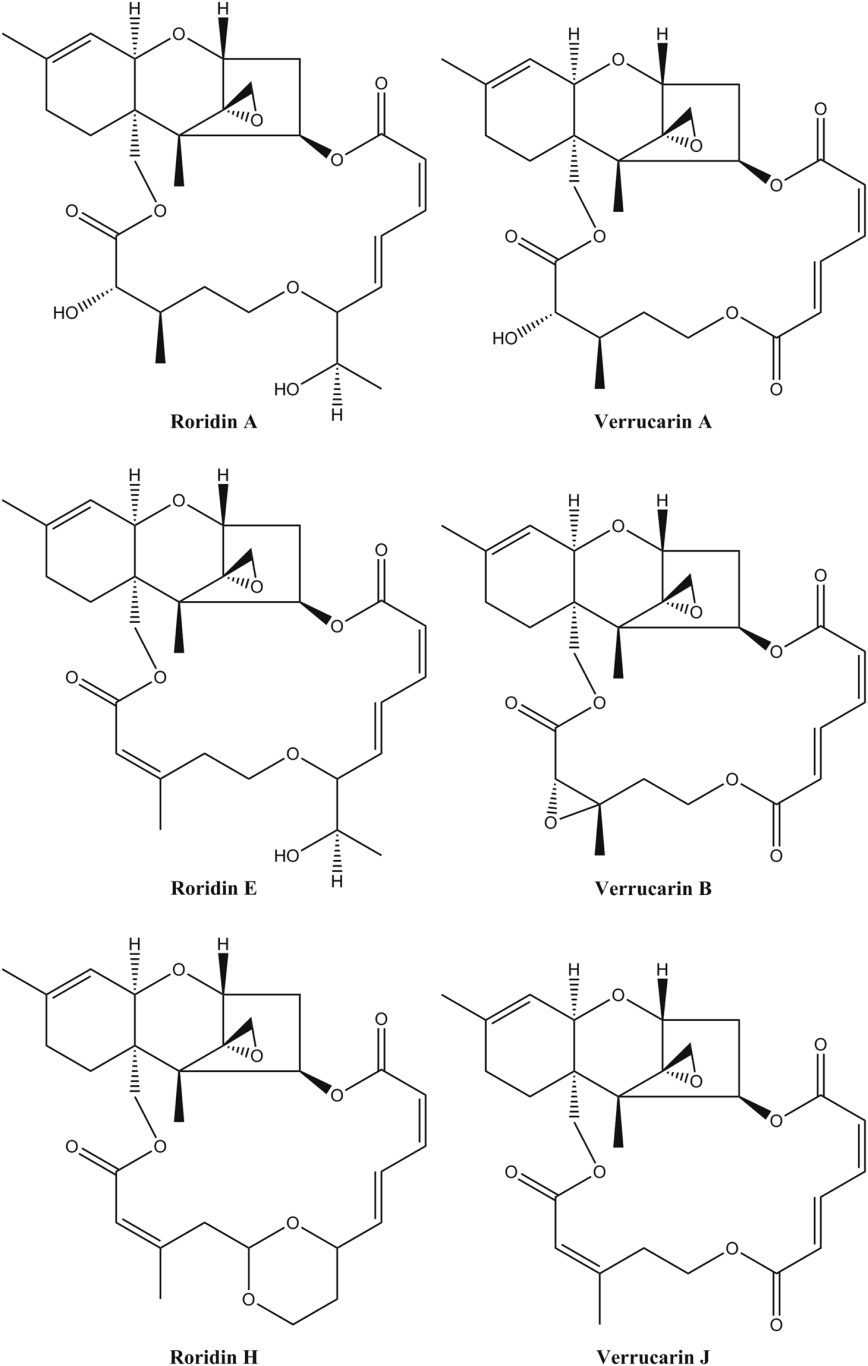
The chemical structures of the family of macrocyclic trichothecenes, which reduce body axis extension (Table 2) show that these compounds clearly belong to the same family of chemical compounds.

The fungal filtrates from 39 species of fungi that induced a reduction in body axis extension were processed to isolate and identify the bioactive compound responsible for the observed defect. We identified 34 known metabolites from 39 bioactive fungi (Table 2, Fig. 5, Supplementary Fig. S1). These known metabolites include known therapeutic compounds such as the antifungal griseofulvin, immunosuppressant cyclosporine and HMG-CoA reductase inhibitors compactin (also known as mevastatin) and lovastatin. Furthermore, a noteworthy compound we found in our screen is bostrycin. This compound has been found to inhibit the PI3K/AKT-pathway in lung tumor cells^31^ and confirmed our hypothesis that inhibitors of this pathway might be among the bioactive compounds. Other identified compounds include sparsely investigated compounds such as anthracobic acid A and CJ-17572. Since not much is known about these compounds and they induced similar phenotypes as therapeutic compounds, they are interesting for further investigation. On the other hand, one group of compounds we repeatedly found was the group of (macrocyclic) trichothecenes (Fig. 6) produced by 11 fungi selected for further investigation. These molecules have a characteristic backbone, containing an epoxy-group at C-12 and are reported to be highly toxic. However, in our screen we found characteristic phenotypes in nanomolar concentrations suggesting that these compounds might act on a specific pathway in low concentrations. All analytical chemical data of the identified compounds is available in the Supplementary Note.

**Table 2:** Secondary metabolites that induce developmental defects in zebrafish^43–47^.

Compound	Phenotype	Isolated from	CBS-number	Ref.
Anthracobic acid A	Reduced body axis extension, less fin formation, heart edema	*Trichophaea abundans*	CBS 305.72	—
Bostrycin	Reduced body axis extension, colored yolk	*Arthrinium pheaospermum*	CBS 142.55	^43^
Brefeldin A*	Reduced body axis extension	*Eupenicillium brefeldianum*	CBS 291.62	^44^
7-dehydrobrefeldin A				
		*Acremonium roseolum*	CBS 446.68	—
		*Acremonium crotocinigenum*	CBS 408.70	—
		*Eupenicillium javanicum*	CBS 448.74	—
		*Coniochaeta angustispora*	CBS 140.79	—
		*Lecanicillium fungicola*	CBS 357.80	—
CJ-17572	Reduced body axis extension, necrosis in high concentration	*Pezicula sporulosa*	CBS 225.96	—
Cladoporin/Asperentin Isocladosporin	Reduced body axis extension	*Venturia crataegi*	CBS 368.35	—
		*Botryotinia sphaerosperma*	CBS 118797	
Compactin/Mevastatin*	Pigmentation, Reduced body axis extension, Blood accumulation	*Absidia cylindrospora*	CBS 127.68	—
Dihydrocompactin		*Penicillium commune*	CBS 427.65	^45^
Cyclosporin A	Reduced body axis extension, heart edema	*Engyodontium album*	CBS 348.55	—
Cyclosporin B				
Fumagillin*	Reduced body axis extension, kink in notochord, dark yolk	*Penicillium scabrosum*	CBS 530.97	^46^
Fusaric Acid*	Undulating notochord	*Fusarium fujikoroi*	CBS 183.29	—
		*Fusarium proliferatum*	CBS 533.95	—
		*Fusarium opheodes*	CBS 118510	—
		*Fusarium sacchari*	CBS 245.59	—
		*Fusarium proliferatum*	CBS 240.64	—
Griseofulvin*	Reduced body axis extension	*Penicillium sp*.	CBS 532.71	—
Dechlorogriseofulvin				
Demethylgriseofulvin				
Macrocyclic trichothecenes:	Truncation, Reduced body axis extension in lower dilutions with heart, yolk and tail; defects	*Myrothecium leucotrichum*	CBS 256.57	—
Roridin A		*Stanjemonium spectable*	CBS 340.70	—
Roridin E		*Gabarnaudia tholispora*	CBS 351.70	—
Roridin H		*Amauroascus kuehnii*	CBS 632.72	—
Verrucarin A*				
Verrucarin B				
Verrucarin J				
Mevinolin/Lovastatin*	Pigmentation, Reduced body axis extension, Blood accumulation	*Resinicium furfuraceum*	CBS 637.78	—
Orsellinic acid	Slight Reduced body axis extension, heart edema	*Exophiala lecanii-corni*	CBS 124176	—
Pseurotin A	Slight Reduced body axis extension, heart edema	*Pseudodiplodia ruticola*	CBS 286.72	—
Pseurotin E		*Pseaudallescheria ellipsoidea*	CBS 418.73	—
Sterigmatocystin*	Finless, less fins	*Emericella foeniculicola*	CBS 156.80	^47^
5,6-dimethoxysterigmatocystin		*Emericella heterothallica*	CBS 489.65	^47^
Methoxysterigmatocystin				
T2-toxin*	Truncation, Reduced body axis extension in lower dilutions with heart, yolk and tail; defects	*Fusarium sporotrichioides*	CBS 413.86	—
Tenuazonic acid*	Reduced body axis extension, heart edema	*Alternaria alternata*	CBS 101.13	—
Trichodermin	Truncation, Reduced body axis extension in lower dilutions with heart, yolk and tail; defects	*Nalanthamala psidii*	CBS 116952	—
Hydroxytrichodermin		*Nalanthamala vermoesenii*	CBS 137.24	—
		*Hypocrea rodmanii*	CBS 124347	—
		*Exophiala lecanii-corni*	CBS 124176	—
		*Stibella flavipes*	CBS 146.81	—
Trichothecin	Truncation, Reduced body axis extension in lower dilutions with heart, yolk and tail; defects	*Dendryphion nanum*	CBS 131.68	—

* = these compounds were validated using their commercially available equivalent.

**Figure 7:**
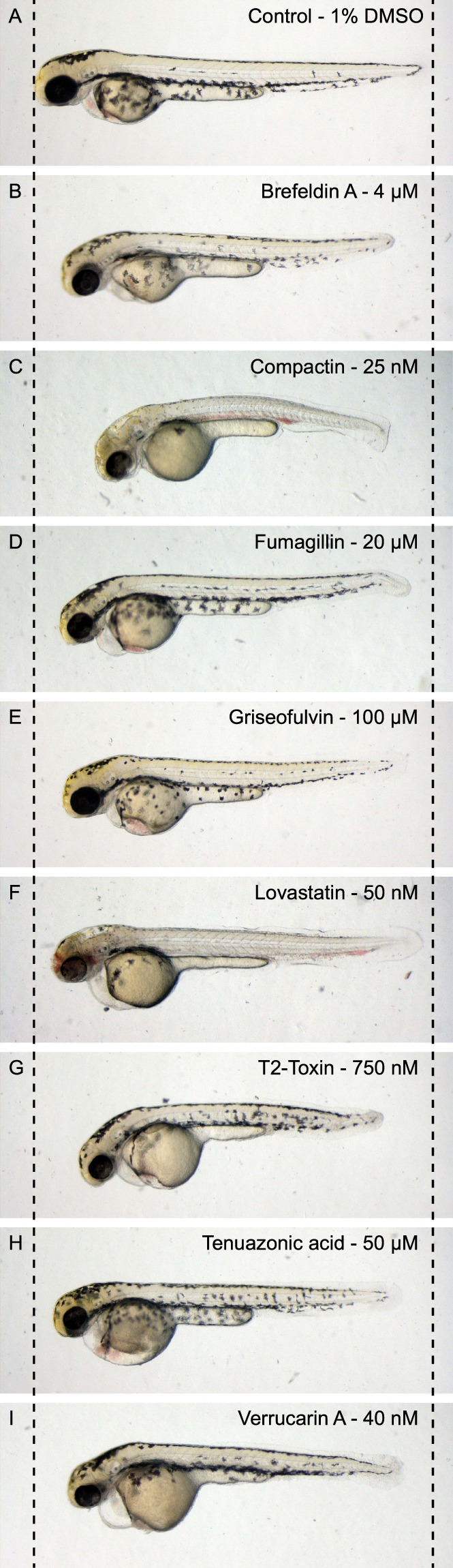
Reduced body axis extension phenotypes induced by commercially available compounds. Embryos were treated with compounds from 6 hpf onwards and were imaged at 48 hpf.

**Figure 8:**
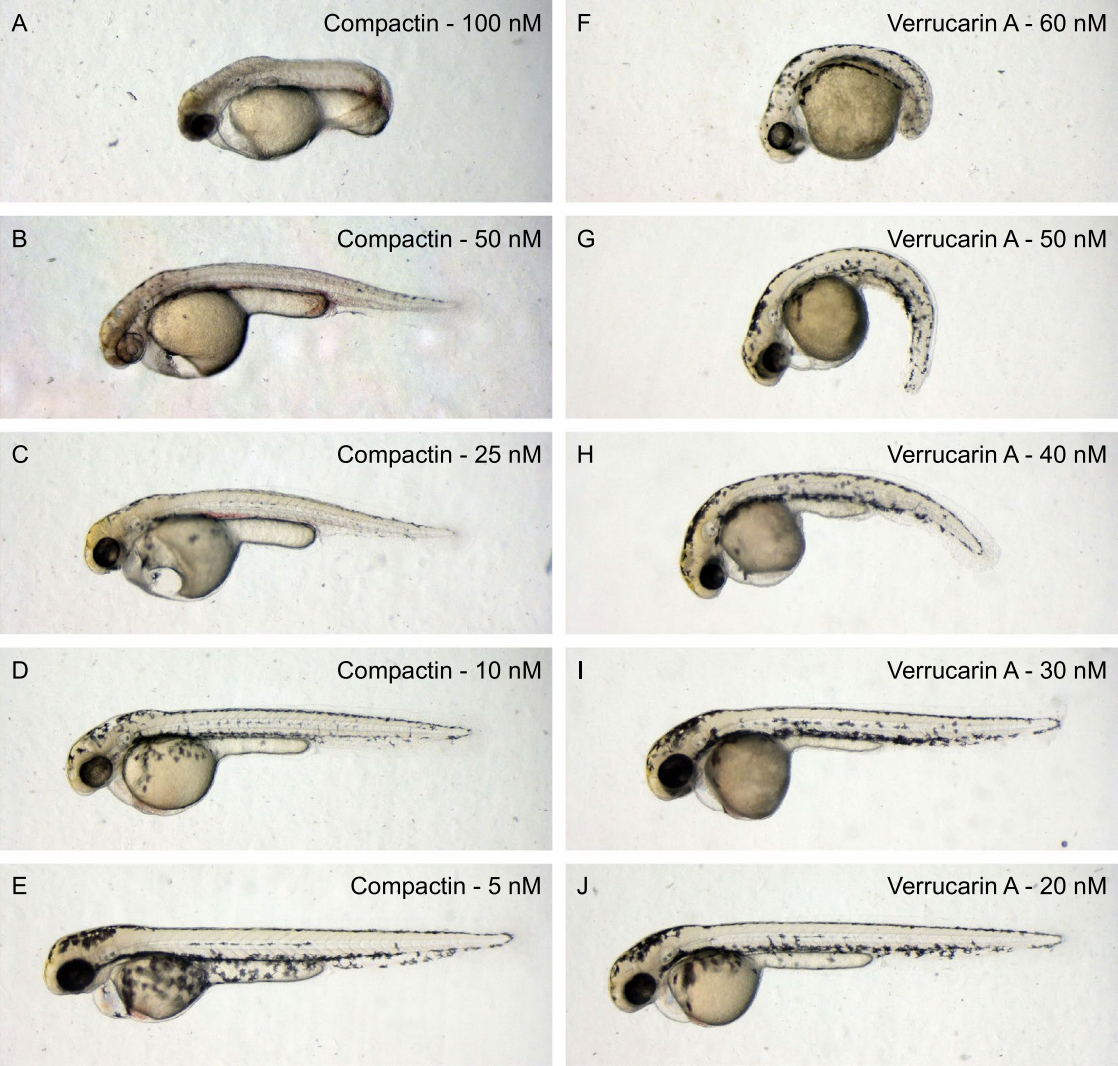
A dilution range reveals specific effects of compactin and verrucarin A on zebrafish embryogenesis. Zebrafish embryos were treated with different concentrations of (**A–E**) compactin or (**F–J**) verrucarin A from 6 hpf onwards and were imaged at 48 hpf. (**A**) 100 nM, (**B**) 50 nM, (**C**) 25 nM, (**D**), 10 nM, (**E**) 5 nM compactin. (**F**) 60 nM, (**G**) 50 nM, (**H**) 40 nM, (**I**) 30 nM, (**J**) 20 nM verrucarin A. Higher concentrations of compactin or verrucarin A were lethal.

**Figure 9:**
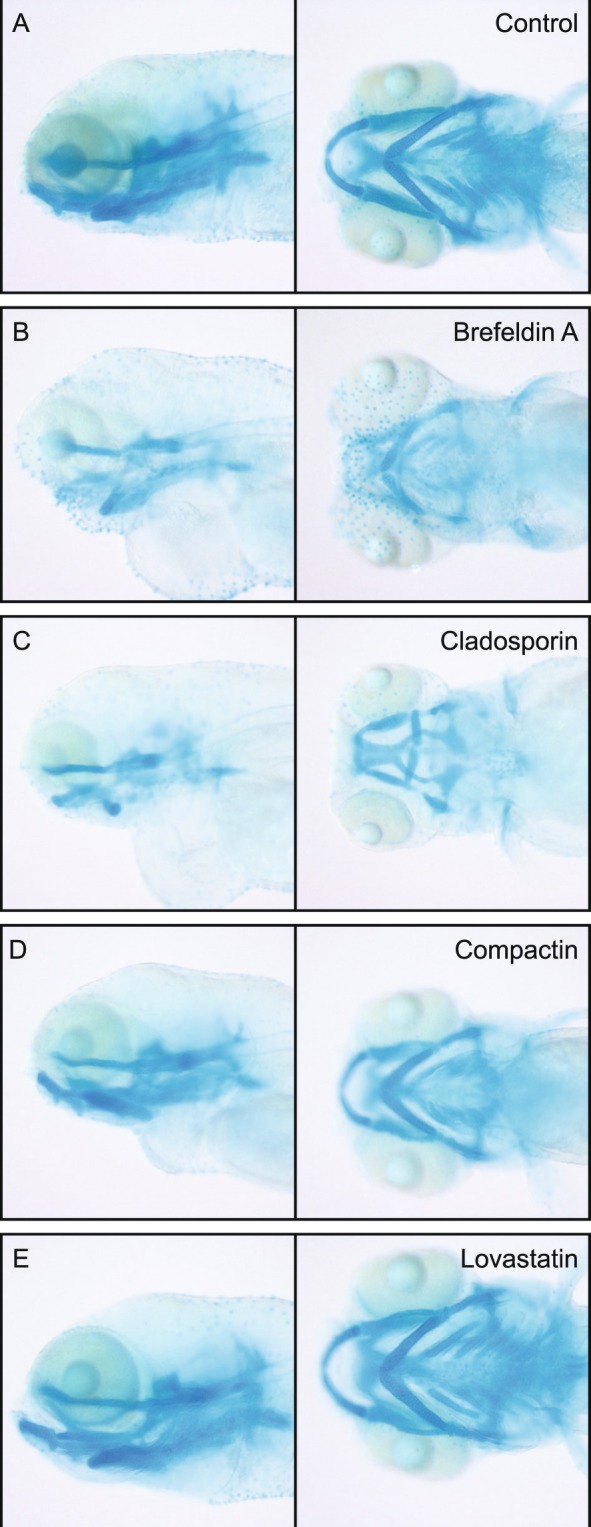
Craniofacial defects in embryos of the “shorter” category. Reduced body axis extension may be due to defects in convergence and extension cell movements, which is accompanied by craniofacial defects. Alcian blue staining was performed and the cartilage was imaged laterally and dorsally to illustrate (**A**) control, and (**B–F**) treated embryos.

To confirm our results, we ordered and tested all commercially available compounds in serial dilutions and found shorter phenotypes in all of them (Fig. 7). In the process, we found additional phenotypes of several compounds in high concentrations. Therapeutic statin compounds, like mevastatin, showed pleiotropic phenotypes including curved tail, reduced pigmentation, cardiac edema and affected vasculature in high concentrations (Fig. 8 − 100 nM compactin). In lower concentrations these severe effects were abolished and the reduced body axis extension phenotype became evident (Fig. 8). Likewise, the trichothecene compounds induced truncation in high concentration (Fig. 8–60 nM verrucarin A). Again, in lower concentrations this effect is abolished and we observed the reduced body axis extension phenotype. Finally, to assess whether embryos in the reduced body axis extension category also displayed characteristic craniofacial defects, we performed alcian blue stainings on compound treated embryos and found that indeed these treated embryos displayed craniofacial defects, including blunted face and wide-set eyes (Fig. 9). These craniofacial defects resemble the defects in embryos lacking functional Shp2^23^, suggesting that specific signaling pathways are inhibited in response to the fungal secondary metabolites. It will be interesting in future work to assess whether these diverse compounds act on the same or distinct signaling pathways in zebrafish embryos.

## Discussion

Here, we describe the identification of biologically active compounds from a library of fungal secondary metabolite mixtures from 10,207 strains of fungi.

The production of secondary metabolites for any given fungus depends heavily on the culture medium and the state of growth. In our approach, we chose CDB + 0,5% yeast extract as medium for yielding bioactive metabolites as it gave the best results in our pilot experiments with a variety of media. Other media might be more suitable for metabolite production for particular fungi and therefore the potential of some fungi may have been missed in this screen. However, overall our method proved to be quite successful as we obtained 1526 bioactive fungal filtrates using the zebrafish embryo assay alone.

Zebrafish embryogenesis is an excellent read-out for biological activity of compounds in vertebrates in medium to high-throughput screens. Zebrafish embryos are transparent and embryogenesis proceeds in a stereotypical manner, facilitating identification of differences between embryos that have been treated with distinct compounds. A great advantage of the zebrafish over cell-based systems is that multiple cell types are present in zebrafish embryos and effects on basically all cell types are tested simultaneously. Moreover, in the set-up we used, bioavailability of the active compounds is assessed at the same time as well. If the fungal mixture contains secondary metabolites that do not penetrate the chorion surrounding the embryo, no effect on embryogenesis will become evident. As a result, it is possible that we missed bioactive secondary metabolites in our screen. However, we have identified a large number of positive hits and the molecular weight of most of the bioactive compounds is below 800 Da, which is an excellent starting point for further development into drugs.

The purification method we developed using liquid-liquid extraction and reversed phase HPLC, proved to be successful for the isolation of a wide variety of active compounds. This method is not suitable for highly polar compounds, because these compounds would not be extracted from the fungal filtrates by ethyl acetate. None of the active compounds we found were highly polar. The amount of analytical chemical data needed for successful identification varied from compound to compound (Supplementary Note). For several compounds, obtaining a monoisotopic mass spectrum in combination with a UV-Vis spectrum and comparing these data to literature^17,18^, was sufficient for compound identification. In other cases, we performed additional NMR measurements to verify the structure. The cyclic peptides cyclosporine A and B were identified based on their MSMS fragmentation profile^32^. Several active compounds were commercially available. We obtained these and verified their activity in the zebrafish bioassay (Table 2)

The purified active compounds induced specific phenotypes in zebrafish embryos, providing insights into the underlying developmental processes. We identified fusaric acid to be the bioactive compound from *Fusarium proliferatum* that induced an undulating notochord. Earlier, Yin *et al*. reported that fusaric acid induces an undulating notochord, which is caused by chelation of copper^21^. The defects can be rescued by addition of exogenous copper, indicating that a minimal concentration of copper ions is required for normal notochord development.

The observed developmental defects may also provide insight into the mode-of-action of the compounds that induce these defects. For instance, in our screen we found that bostrycin caused a similar phenotype as the small molecule PI3K inhibitor, LY294002. Bostrycin has also been reported to inhibit the PI3K/Akt signaling pathway^31^ and both compounds are reported to have cancer suppressing properties. Therefore, it is likely that bostrycin exerts its effects on zebrafish development by inhibition of PI3K signaling. Likewise, other less studied compounds that cause similar defects might also have a similar mode-of-action. For instance, in our assay we identified relatively unexplored secondary metabolites such as CJ-17572 and anthracobic acid A, to cause reduced body axis extension as well. The information on the bioactivity of these compounds is sparse. The only paper that has been published to date about anthracobic acid A reported it to have antimicrobial activity^33^. Perhaps anthracobic acid also has an effect on PI3K signaling, resulting in reduced body axis extension in zebrafish embryos.

The macrocyclic trichothecenes have been reported to affect Mitogen Activated Protein Kinase (MAPK) signaling. Different trichothecenes activate Jun kinase (JNK), p38 MAPK and extracellular signal-regulated protein kinase (ERK) to different extents in murine RAW 264.7 macrophage and U937 human leukemic cells^34^. Moreover, the trichothecene, Verrucarin A, inhibited phorbol ester-induced JNK, p38 MAPK and ERK activation^35^. Both inhibition and activation of the RAS/MAPK signaling pathway in zebrafish embryos affected convergence and extension cell movements, resulting in reduced body axis extension^25,36,37^. Together, these results suggest that the macrocyclic trichothecenes may affect zebrafish development by activation or inhibition of MAPK signaling. Future work on the working mechanism of trichothecenes will reveal how they actually affect zebrafish embryonic development.

A large number of small molecule screens using FDA-approved drugs and small molecule inhibitors have been carried out using zebrafish embryos to date^13^. The working mechanism of an increasing number of known small molecule compounds is being elucidated. Comparison of the phenotypical defects of these compounds in zebrafish with the phenotypic defects caused by lesser known compounds from our fungal secondary metabolite library may provide clues to their working mechanisms. Moreover, knowledge about their mode-of-action may accelerate development of these fungal compounds into lead compounds and beyond into therapeutics.

One category of phenotypes that is very promising for identification of novel therapeutics is the pigmentation defects category. Factors involved in melanocyte formation also have a crucial role in melanoma formation and progression^38^. In several studies, compounds that reduce pigmentation in zebrafish embryos have been found to inhibit melanoma progression. One major example is the compound leflunomide, which is used for treatment of arthritis in human patients. Leflunomide is found to inhibit melanocyte formation in zebrafish embryos and is currently being tested in a clinical trial as treatment for melanoma^39^. Likewise, the therapeutic statin compounds, compactin and lovastatin, which reduced pigmentation when administered in high concentrations in our screen, were also found to inhibit proliferation and invasion of melanoma cells in high concentration^40^. Finally, in a compound screen performed by Colanesi *et al*.^41^. over 50 therapeutic compounds were found to affect pigmentation in zebrafish embryos. We believe there is a great potential in fungal filtrates, which cause reduced pigmentation, as they may lead to identification of compounds for future development into drugs to combat melanoma. Currently, we are purifying compounds from this category in an attempt to establish the identity of active compounds.

Taken together, the library of fungal mixtures that we generated expresses a diverse array of biologically active compounds, of which we have explored only a small part. This library is likely to contain many more distinct biologically active compounds. In fact, the library can be used in practically any assay for biological activity and therefore represents a great resource to uncover biologically active compounds.

## Materials and Methods

### Generation of fungal filtrate library

Lyophilized fungal strains, provided by the Westerdijk Fungal Biodiversity Institute fungal collection (Utrecht, the Netherlands), were inoculated and grown for 7 days on agar plates preferential for the respective fungal species, according to instructions provided. In cases of poor fungal growth, the incubation time was extended. Subsequently, cubes of approximately 5 × 5 mm were cut from each agar plate. Two cubes per fungus were used to inoculate a 100 mL bottle containing 50 mL medium (3.5% Czapek Dox Broth + 0.5% Yeast extract). Remaining cubes were used for long term storage. The inoculated broth was incubated at RT for 7 days. The medium was then filter sterilized using a 0.22 µm Millipore filter. These fungal filtrates were stored at −20 °C before testing.

### Zebrafish embryo assay

Zebrafish eggs obtained from family crosses of Tuebingen Long fin zebrafish lines were used to assess biological activity. Prior to treatment with fungal filtrate the zebrafish eggs were bleached 2 × 5 minutes with sodium hypochlorite (10–13% active chloride, 0.36 mL/L) in E3-medium and washed extensively with E3 medium. Subsequently, the embryos were kept in E3 medium supplemented with 0.2% penicillin/streptomycin to prevent infections with microorganisms. The eggs were then divided over 24-well plates, 5 embryos per well in 500 µL E3-medium + antibiotics (penicillin and streptomycin). At 6hpf, 500 µL of fungal filtrate was added to each well. At 24 hpf and 48 hpf, the zebrafish embryos were inspected for morphological developmental defects. Filtrates were scored as positive if 3 or more embryos were affected. Positively scored fungal filtrates were tested in serial dilutions until an ineffective dilution was reached. Morphological defects were imaged using a Leica MZFLIII microscope equipped with a Leica DFC320 camera.

All procedures involving experimental animals were approved by the local animal experiments committee (Koninklijke Nederlandse Akademie van Weterschappen-Dierexperimenten commissie) and performed according to local guidelines and policies in compliance with national and European law. Adult zebrafish were maintained as previously described^42^.

### Purification of biologically active compounds

Fungi selected for activity-guided purification were grown as described above. To obtain a larger volume of fungal filtrate, multiple 100 mL bottles, each with 50 mL medium, were inoculated. After filtration, the fungal filtrates were extracted with 3 × 1/3 volume (equal to volume of fungal filtrate) ethyl acetate using a separation funnel. The ethyl acetate layer was collected and evaporated to dryness using a rotary evaporator with the water bath temperature set at 40 °C. The residue was dissolved in 1 mL DMSO. Next, the extract was tested for its activity by incubation with zebrafish embryos in E3 medium without antibiotics to establish whether biologically active component(s) were extracted successfully. Positively tested extracts were then fractionated on a preparative Shimadzu LC-MS QP8000 HPLC system using a Dr. Maisch C18 Reprosil-AQ column (10 µm, 120 Å, 250 × 22 mm) and a Shimadzu SPD-10A UV-detector set at 214 nm and 254 nm. The mobile phase was 0.1% trifluoroacetic acid in acetonitrile:water 5:95 (buffer A) and 0.1% trifluoroacetic acid in acetonitrile:water 95:5 (buffer B). A flow rate of 12.5 mL min-1 was applied using the following protocol: 100% buffer A for 5 minutes followed by a linear gradient of buffer B (0–100%) for 80 minutes, 100% buffer B for 5 minutes, another linear gradient of buffer B (100–0%) for 5 minutes and finally 100% buffer A for 5 minutes. Fractions were collected every 63 seconds, resulting in 95 fractions of 13 mL. 1 ml of each collected fraction was dried in a speed-vac overnight and dissolved in DMSO. Next, fractions were tested for their activity. Active fractions have been analyzed using analytical chemical methods as described below.

### Identification of biologically active compounds

Firstly, active fractions were assessed for their purity through analytical HPLC, either using a Shimadzu LC-2030 system with PDA detection (190–800 nm) or a Shimadzu SCL-10A system with UV detection at 214 and 254 nm (Shimadzu SPD-10A). Simultaneously, the PDA detection of the first system allows a UV-Vis spectrum to be obtained for each compound. UV-Vis spectra for compounds measured on the latter HPLC system were obtained using a Thermo TEC-UV1 v.4.60 UV-Vis spectroscope (190–800 nm with 1 nm scan interval). Secondly, LC-MS spectra were measured on a Finnigan LCQ Deca XP Max system in positive ionization mode, followed by more accurate high resolution mass spectrometry (HRMS) and tandem mass spectrometry measured on a µQTOF instrument (Micromass Ltd, Manchester UK). The sample was mixed with sodium formate for HRMS resulting in the detection of sodium adduct ions. This procedure resulted in an internal calibrant in each sample and facilitated identification of the more accurate mass of the compounds. The obtained MS and UV data were compared with literature data and proved to be sufficient to identify the active components from several active fractions. Other fractions required extensive NMR measurements for identification. For this purpose, the remainders of the active fractions were dried in a speedvac and dissolved in 400 mL DMSO-d6. Next, ^1^H-NMR spectra were measured at either 300 MHz, 400 MHz or 500 MHz using either a Mercury-300, an Agilent-400 or an INOVA-500 spectrometer respectively. The^13^C-NMR spectrum for Anthracobic acid A was obtained at 100 MHz using an INOVA-500 spectrometer. Furthermore, for the identification of Anthracobic acid, we performed 2D-NMR experiments, including Correlation Spectroscopy (COSY), Total Correlation Spectroscopy (TOCSY), Heteronuclear Single-Quantum Correlation Spectroscopy (HSQC) and Heteronuclear Multiple-Bond Correlation spectroscopy (HMBC).

### Alcian blue

Embryos were treated with compound from 6 hpf until 4 dpf. Embryos were then anesthetized with tricane mesylate and fixed in 4% paraformaldehyde overnight at 4 °C. Subsequently, embryos were washed in 50% ethanol for 10 minutes. Embryos were stained overnight in staining solution (0.04% Alcian Blue, 70% Ethanol and 50 mM MgCl_2_) at 4 °C. Finally, embryos were bleached using bleaching solution (8.5% hydrogen peroxide, 5% formamide, 0.5% SSC) and imaged in 80% glycerol supplemented with 1% KOH using Leica MZFLIII microscope equipped with a Leica DFC320 camera. Images were analyzed using ImageJ.

## Supplementary information

Supplementary Material

Supplementary Table S1
