# Supplementary material for: A new perspective on fungal metabolites: identification of bioactive compounds from fungi using zebrafish embryogenesis as read-out
Source: Sci Rep. 2019 Nov 26;9:17546. doi: 10.1038/s41598-019-54127-9 (PMC6879544; doi:10.1038/s41598-019-54127-9)
Supplement: Supplementary file 1 — Supplementary Material [file 41598_2019_54127_MOESM1_ESM.pdf]

Supplementary material

Title: A new perspective on fungal metabolites: identification of bioactive compounds from fungi using zebrafish embryogenesis as read-out

Jelmer Hoeksma, Tim Misset, Christie Wever, Johan Kemmink, John Kruijtzer, Kees Versluis, Rob M.J. Liskamp, Geert Jan Boons, Albert J.R. Heck, Teun Boekhout, Jeroen den Hertog

Supplementary Figure S1. Reduced body axis extension phenotypes induced by identified compounds.

Supplementary Note. Analytical chemical data of identified compounds.

Supplementary Table S1. List of 10,207 fungi that were used to generate the library in alphabetical order

(.xlsx file under separate cover).

Supplementary Figure S1

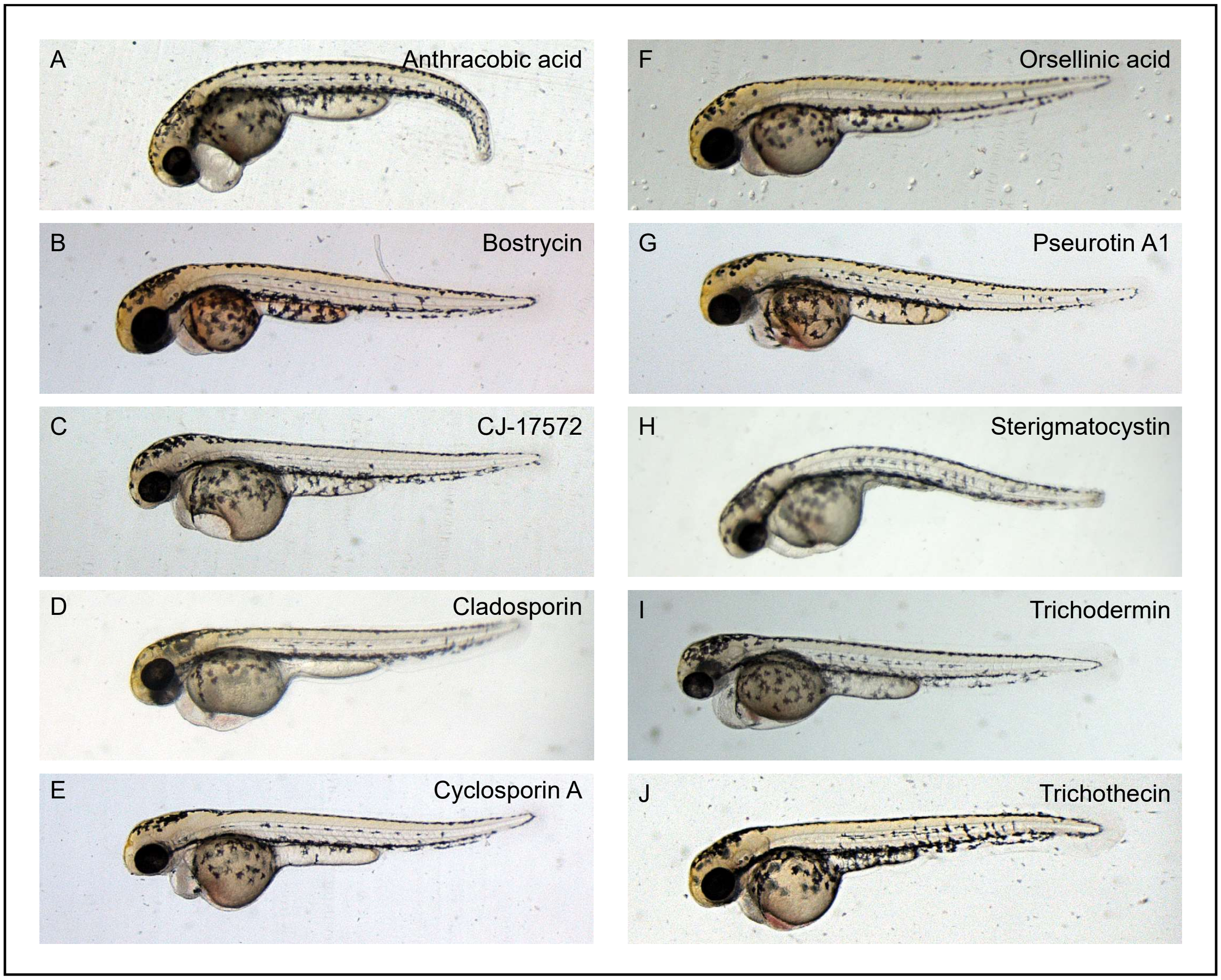

Supplementary Figure S1. Reduced body axis extension phenotypes induced by identified compounds. Embryos were treated with compounds from 6 hpf onwards and were imaged at 48 hpf.

Supplementary note

Spectral data of identified compounds

Anthracobic acid A: $C_{25}H_{32}O_4$. HRMS: found 419.2213 (M+Na), calculated 419.2198 for $C_{25}H_{32}O_4Na$. MSMS fragmentation: 379(base), 361, 228 m/z. ^{1}H-NMR (500 MHz, d^6-DMSO): δ= 7.24 (dd, 1H, *J*=11.5, 14,9Hz); 6.72 (d, 1H, *J*=11.3Hz); 6.54 (dd, 1H, *J*=11.8, 14.3Hz); 6.45 (dd, 1H, *J*=11.8, 14.3Hz); 6.27 (d, 1H; *J*=11.3Hz); 5.90 (d, 1H, *J*=14.8Hz); 5.40 (m, 1H); 5.37 (m, 1H); 5.30 (s, 1H); 3.12 (d, 1H); 3.00 (d, 1H); 2.15 (m, 1H); 2.11, 1.92 (m, 2H); 1.85 (s, 3H); 1.68, 1.38 (m, 2H); 1.64 (s, 3H); 1.63 (s, 3H); 1.60 (m, 1H); 1.57 (s, 3H); 1.37 (1H). ^{13}C-NMR (100 MHz, d^6-DMSO): δ= 168.5; 145.4; 145.1; 138.6; 138.1; 137.8; 135.4; 133.0; 129.0; 125.4; 121.6; 121.4; 120.9; 76.3; 60.7; 57.9; 50.4; 36.6; 35.6; 34.8; 30.9; 22.7; 14.1; 13.2; 11.9. UV λ_{max}: 204 nm, 240 nm, 340 nm. Data consistent with data published by Shiono [1].

Bostrycin: $C_{16}H_{16}O_8$. HRMS: found 359.0757 (M+Na), calculated 359.0743 for $C_{16}H_{16}O_8Na$. MSMS fragmentation: 337(base), 319, 301, 273, 167 m/z. UV λ_{max}: 212 nm, 228 nm, 307 nm, 476 nm(sh), 504 nm, 538 nm(sh). Data consistent with data published by Nielsen and Smedsgaard [2].

Brefeldin A: $C_{16}H_{24}O_4$. HRMS: found 303.1568 (M+Na), calculated 303.1572 for $C_{16}H_{24}O_4Na$. MSMS fragmentation: 281(base), 263, 245, 199, 163 m/z. UV λ_{max}: 216 nm (broad peak).
7-dehydrobrefeldin A: $C_{16}H_{22}O_4$. LC-MS (ESI): found 279.0 (M+H). MSMS fragmentation: 279(base), 261, 243. UV λ_{max}: 200 nm. Data consistent with data published by Nielsen and Smedsgaard[2]. Brefeldin A confirmed by commercially available compound.

CJ-17572: $C_{21}H_{31}NO_4$. HRMS: found 384.2141 (M+Na), calculated 384.2153 for $C_{21}H_{31}NO_4Na$. MSMS fragmentation: 362(base), 344, 318, 184, 149 m/z. ^{1}H-NMR (300 MHz, $CDCl_3$): δ= 5.52(1H); 5.34 (d, 1H, *J*=9.7Hz); 4.20 (1H); 3.79 (1H); 2.99 (3H); 1,78 (mH)*; 1.46 (4H)*; 1.26 (3H); 1.08 (1H); 1.07 (1H); 0.93 (3H); 0.84 (3H). UV λ_{max}: <200 nm, 235 nm, 294 nm. *overlapping peaks. Data consistent with data of Sugie *et al.*[3].

Cladosporin: $C_{16}H_{20}O_5$. HRMS: found 315.1213 (M+Na), calculated 315.1208 for $C_{16}H_{20}O_5Na$. MSMS fragmentation: 293(base), 275, 257, 99 m/z. UV λ_{max}: 216 nm, 268 nm, 300 nm(sh)..
Isocladosporin: $C_{16}H_{20}O_5$. HRMS: found 315.1209 (M+Na), calculated 315.1208 for $C_{16}H_{20}O_5Na$. UV λ_{max}: 216 nm, 268 nm, 300 nm(sh). Data consistent with data published by Nielsen and Smedsgaard[2].

Compactin/Mevastatin: $C_{23}H_{34}O_5$. HRMS: found 413.2312 (M+Na), calculated 413.2304 for $C_{23}H_{34}O_5Na$. MSMS fragmentation: 391(base), 271, 253, 229, 185. UV λ_{max}: No max found.
Dihydrocompactin/Mevastatin: $C_{23}H_{36}O_5$. HRMS: found 415.2475 (M+Na), calculated 415.2460 for $C_{23}H_{34}O_5Na$. UV λ_{max}: No max found. Data consistent with data published by Nielsen and Smedsgaard[2], Compactin confirmed by commercially available compound.

Cyclosporin A: $C_{62}H_{111}N_{11}O_{12}$. LCMS(ESI): 1203.2 (M+H). MSMS fragmentation: 1203, 675, 425, 397, 298, 224, 199.
Cyclosporin B: $C_{61}H_{109}N_{11}O_{12}$. LCMS(ESI): 1189.2 (M+H). MSMS fragmentation: 1189, 661, 425, 397, 298, 224.

MSMS data consistent with data of Bowers *et al.* [4]. Cyclosporin A confirmed by commercially available compound.

Fumagillin: $C_{26}H_{34}O_7$. HRMS: found 481.2193 (M+Na), calculated 481.2203 for $C_{26}H_{34}O_7Na$. UV λ_{max}: 335 nm, 351 nm. Data consistent with data published by Nielsen and Smedsgaard[2], and Garrett and Elbe[5]. Confirmed by commercially available compound.

Fusaric Acid: $C_{10}H_{13}NO_2$. LC-MS (ESI): found 180.1 (M+H). ^{1}H-NMR (400 MHz, d^6-DMSO): δ= 8.27 (s, 1H); 7.99 (d, 1H, *J*=7.9Hz); 7.85 (d, 1H, *J*=7.8Hz); 2,69 (t, 2H); 1.58 (m, 2H); 1,31 (dd, 2H); 0.9 (t, 3H). UV λ_{max}: 202 nm, 225 nm, 270 nm. Data consistent with data published by Nielsen and Smedsgaard [2]. Confirmed by commercially available compound.

Griseofulvin: $C_{17}H_{17}ClO_6$. HRMS: found 375.0611(M+Na), calculated 375.0607 for $C_{17}H_{17}ClO_6Na$. UV λ_{max}: 214 nm, 236 nm, 294 nm, 324 nm(sh). Confirmed by commercially available compound.
Dechlorogriseofulvin: $C_{17}H_{18}O_6$. LCMS (ESI): found 319.0 (M+H). UV λ_{max}: 253 nm, 287 nm.
Demethylgriseofulvin: $C_{16}H_{15}ClO_6$. LCMS (ESI): found 339.1 (M+H). UV λ_{max}: 212 nm, 233 nm, 288nm. Data consistent with data published by Nielsen and Smedsgaard[2]. Griseofulvin confirmed by commercially available compound.

<u>Macrocylic trichothecenes:</u>
Roridin A: $C_{29}H_{40}O_9$. HRMS: found 555.2542 (M+Na), calculated 555.2570 for $C_{29}H_{40}O_9Na$. MSMS fragmentation: 533 (base), 406, 333, 249. UV λ_{max}: 262 nm.
Roridin E: $C_{29}H_{38}O_8$. HRMS: found 515.2618 (M+H), calculated 515.2645 for $C_{29}H_{39}O_8^+$. MSMS fragmentation: 515 (base), 361, 231. UV λ_{max}: 224 nm, 263 nm.
Roridin H: $C_{29}H_{36}O_8$. HRMS: found 535.2310 (M+Na), calculated 535.2308 for $C_{29}H_{36}O_8Na$. MSMS fragmentation: 513 (base), 401, 341, 327.
Verrucarin A: $C_{27}H_{34}O_9$. HRMS: found 525.2087 (M+Na), calculated 525.2101 for $C_{27}H_{34}O_9Na$. MSMS fragmentation: 503 (base), 457, 373, 249, 231. UV λ_{max}: 259 nm.
Verrucarin B: $C_{27}H_{32}O_9$. HRMS: found 523.1880 (M+Na), calculated 523.1885 for $C_{27}H_{34}O_9Na$. MSMS fragmentation: 501 (base), 249, 231. UV λ_{max}: 261 nm.
Verrucarin J: $C_{27}H_{32}O_8$. HRMS: found 485.2150 (M+H), calculated 485.2175 for $C_{27}H_{32}O_8^+$. MSMS fragmentation: 485 (base), 373, 343, 249, 231. UV λ_{max}: 262 nm.
Data consistent with data published by Nielsen and Smedsgaard[2]. Verrucarin A confirmed by commercially available compound.

Mevinolin/Lovastatin: $C_{24}H_{36}O_5$. HRMS: found 427.2463 (M+Na), calculated 427.2460 for $C_{24}H_{36}O_5Na$. MSMS fragmentation: 405(base), 303, 285, 243, 199. UV λ_{max}: 231 nm(sh), 239, 247(sh) . Data consistent with data published by Nielsen and Smedsgaard [2]. Confirmed by commercially available compound

Orsellinic acid: $C_8H_8O_4$. HRMS: found 191.0328 (M+Na), calculated 191.0320 for $C_8H_8O_4Na$. ^{1}H-NMR (400 MHz, d^6-DMSO): δ= 6.17(s, 1H); 6.12(s, 1H); 2.39(s, 3H). UV λ_{max}: 218 nm, 262 nm, 298 nm. Data consistent with data published by Nielsen and Smedsgaard [2], and Sanchez et al. – 2010[6].

Pseurotin A_1: $C_{22}H_{25}NO_8$. HRMS: found 454.1463(M+Na), calculated 454.1478 for $C_{22}H_{25}NO_8Na$. MSMS fragmentation: 432(base), 316. ^{1}H-NMR (300 MHz, d^6-DMSO)*: δ= 9.93 (s, 1H); 8.23 (d, 2H); 7.66 (t, 1H); 7.51 (t,2H); 5.36 (d,2H), 4.43-4.33 (m,3H), 2.02 (m,3H), 1.61 (s,3H), 0.86 (t,3H). UV λ_{max}: 202 nm, 257 nm, 282 nm(sh).
Pseurotin A_2: $C_{22}H_{25}NO_8$. HRMS: found 454.1473(M+Na), calculated 454.1478 for $C_{22}H_{25}NO_8Na$. UV λ_{max}: 203 nm, 258 nm, 282 nm(sh).
Pseurotin E: $C_{22}H_{23}NO_9$. LCMS(ESI): found 467.4(M+Na); 911(2M+H).
Data consistent with data published by Wang *et al.*[7], Breitenstein *et al.*[8], and Schmeda-Hirshmann *et al.*[9].

Sterigmatocystin: $C_{18}H_{12}O_6$. LCMS (ESI): found 325.2 (M+H). UV λ_{max}: 200 nm, 245 nm, 326 nm.
Confirmed by commercially available compound
5,6-dimethoxysterigmatocystin: $C_{20}H_{16}O_8$. LCMS (ESI): found 385.2. UV λ_{max}: 200 nm, 250 nm, 334 nm.
5-Methoxysterigmatocystin: $C_{20}H_{16}O_8$. LCMS (ESI): found 355.1. UV λ_{max}: 200 nm, 245 nm, 330 nm.
Data consistent with data published by Nielsen & Smedsgaard[2], Cole *et al.*[10] and Zalar *et al.*[11].

T2-Toxin: $C_{24}H_{39}O_9$. LCMS (ESI): found 489.3(M+Na), 484.3(M+NH_4), 467.3(M+H). UV λ_{max}: 200 nm. Data consistent with data published by Nielsen and Smedsgaard[2]. Confirmed by commercially available compound.

Tenuazonic acid: $C_{10}H_{15}NO_3$. HRMS: found 198.1108 (M+H), calculated 198.1130 for $C_{10}H_{16}NO_3^+$. UV λ_{max}: 220 nm, 279 nm. Data consistent with data published by Nielsen and Smedsgaard[2]. Confirmed by commercially available compound.

Trichodermin: $C_{17}H_{24}O_4$. HRMS: found 315.1572 (M+Na), calculated 315.1572 for $C_{17}H_{24}O_4Na$. MSMS fragmentation: 333(base), 261, 247, 229. ^{1}H-NMR (300 MHz, d^6-DMSO): δ=5.46 (dd, 1H, *J*=8.2, 3.3Hz); 5.26 (s, 1H); 3.62 (d, 1H);3.53(d, 1H);2.99(d, 1H);2.75(d, 1H);2.40-1.76(m,6H); 1.97(d, 3H); 1.61(s, 3H);0.80(s, 3H);0.56(s, 3H). UV λ_{max}: 200nm.
Hydroxytrichodermin: $C_{17}H_{24}O_5$. HRMS: found 331.1530 (M+Na), calculated 331.1521 for $C_{17}H_{24}O_5Na$. MSMS fragmentation: 309(base), 231, 213. UV λ_{max}: 203nm. Data consistent with data published by Cole *et al.*[10].

Trichothecin: $C_{19}H_{24}O_5$. HRMS: found 355.1514 (M+Na), calculated 355.1521 for $C_{19}H_{24}O_5Na$. MSMS fragmentation: 333(base), 261, 247, 229. ^{1}H-NMR (300 MHz, d^6-DMSO): δ= 6.53 (d, 1H); 6.41 (dd, 1H, *J*=10.8, 7.2Hz); 5.77 (d, 1H, *J*=11.3Hz); 5.54 (d, 1H); 4.04 (d, 1H); 3.82 (d, 1H); 3.05 (s, 1H); 2.87 (s, 1H); 2.75 (d, 1H); 2.58 (dd, 1H); 2.17 (d, 1H); 2.05 (d, 3H); 1.89 (d, 1H), 1.69 (s, 3H), 0.94 (s, 3H), 0.57 (s, 3H). Data consistent with data published by Cole *et al.*[10].

1. Shiono Y. Anthracobic Acids A and B , Two Polyketides , Produced by an Endophytic Fungus Anthracobia sp . *Chem Biodivers*. 2006;3:217-223.

2. Nielsen KF, Smedsgaard J. Fungal metabolite screening: Database of 474 mycotoxins and fungal metabolites for dereplication by standardised liquid chromatography-UV-mass spectrometry methodology. *J Chromatogr A*. 2003;1002(1-2):111-136. doi:10.1016/S0021-9673(03)00490-4.

3. Sugie Y, Dekker KA, Inagaki T, et al. A novel antibiotic CJ-12,572 from a fungus, Pezicula sp. *J Antibiot (Tokyo)*. 2002;55(1).

4. Bowers LD, Norman DD, Yan XX, Scheeler D, Carlson KL. Isolation and structural identification of 9hydroxy-9desmethyl-cyclosporine. *Clin Chem*. 1990;36(11):1875-1879.

5. Garrett BER, Eble TE. Studies on the Stability of Fumagillin. *J Am Pharm Assoc (Scientific Ed*. 1954;43(7):385-390. doi:10.1002/jps.3030430702.

6. Sanchez JF, Chiang Y-M, Szewczyk E, et al. Molecular genetic analusis of the orsellinic acid/F9775 gene cluster of Aspergillus nidulans. *Mol Biosyst*. 2010;6(3):587-593. doi:10.1039/b904541d.

7. Wang F, Li D, Zhu T, Zhang M, Gu Q. Pseurotin A 1 and A 2 , two new 1-oxa-7- azaspiro [4 . 4] non-2-ene-4 , 6-diones from the holothurian-derived fungus Aspergillus fumigatus. *Can J Chem*. 2011;89:72-76. doi:10.1139/V10-157.

8. Breitenstein W, Chexal KK, Mohr P, Tamm C. Pseurotin B, C, D, and E. Further new metabolites of Pseudeurotium ovalis Stolk. *Helv Chim Acta*. 1981;64(2):379-388.

9. Schmeda-Hirschmann G, Hormazabal E, Rodriguez JA, Theoduloz C. Cycloaspeptide A and pseurotin A from the endophytic fungus *Penicillium janczewskii*. *Z Naturforsch C Bio Sci*. 2008;63(5-6):383-388.

10. Cole RJ, Schweikert MA, Jarvis BB. *Handbook of Secondary Fungal Metabolites, 3-Volume Set.* Elsevier; 2003.

11. Zalar P, Frisvad JC, Gunde-Cimerman N, Varga J, Samson RA. Four new species of Emericella from the Mediterranean region of Europe. *Mycologia*. 2008;100(5):779-795. doi:10.3852/08-078.
